# HDAC8 Prevents Anthrax Lethal Toxin-induced Cell Cycle Arrest through Silencing PTEN in Human Monocytic THP-1 Cells

**DOI:** 10.3390/toxins9050162

**Published:** 2017-05-16

**Authors:** Soon-Duck Ha, Woohyun Cho, Sung Ouk Kim

**Affiliations:** Department of Microbiology and Immunology, The University of Western Ontario, London, ON N6G 2V4, Canada; sha3@uwo.ca (S.-D.H.); wcho8@uwo.ca (W.C.)

**Keywords:** anthrax lethal toxin, HDAC8, PTEN, JMJD3, cell cycle arrest, macrophage

## Abstract

Anthrax lethal toxin (LeTx) is a cytotoxic virulence factor that causes cell cycle arrest and cell death in various cell types. However, susceptibility to the cytotoxic effects varies depending on cell types. In proliferating monocytes, LeTx has only transient cytotoxic effects due to activation of the phosphoinositide 3-kinase (PI3K)-AKT-mediated adaptive responses. To date, the mechanism of LeTx in activating PI3K-AKT signaling axis is unknown. This study shows that the histone deacetylase 8 (HDAC8) is involved in activating PI3K-AKT signaling axis through down-regulating the phosphatase and tensin homolog 1 (PTEN) in human monocytic THP-1 cells. The HDAC8-specific activator TM-2-51 and inhibitor PCI-34051 enhanced and prevented, respectively, AKT activation and cell cycle progression in LeTx-treated cells. Furthermore, HDAC8 induced tri-methylation of histone H3 lysine 27 (H3K27me3), which is known to suppress PTEN expression, through at least in part down-regulating the H3K27me3 eraser Jumonji Domain Containing (JMJD) 3. Importantly, the JMJD3-specific inhibitor GSK-J4 induced AKT activation and protected cell cycle arrest in LeTx-treated cells, regardless the presence of HDAC8 activity. Collectively, this study for the first time demonstrated that HDAC8 activity determines susceptibility to cell cycle arrest induced by LeTx, through regulating the PI3K-PTEN-AKT signaling axis.

## 1. Introduction

Anthrax lethal toxin (LeTx) is a key virulence factor of *Bacillus anthracis*, the causative agent of anthrax [1]. It comprises the intracellular transporter protective antigen (PA) and the metalloprotease lethal factor (LF), where LF cleaves and inactivates the mitogen-activated protein kinase (MAPK) kinases (MEK) 1 to 6, except 5 [2,3]. Inactivation of MEKs results in an almost complete inactivation of the extracellular signal-regulated kinases (ERKs) and p38 MAPKs, but partial or no effects on c-Jun N-terminal kinases [3,4,5,6]. Inactivation of ERKs and p38 MAPKs leads to cell cycle arrest and cell death in both immune and non-immune cells [4,7,8,9,10,11,12,13,14,15,16]. However, the extent of cytotoxicity elicited by LeTx is dependent on cell types, differentiation stages and their capacity in activating adaptive responses [4,14,15,16]. In human macrophages, inhibition of ERKs by LeTx leads to cell cycle arrest at G_0/1_ phase due to depletion of cyclin D1 [15], which is required for cell cycle progress from G_1_ to S phase [17]. In contrast, cells with constitutively active or adaptively activating the PI3K-AKT (also known as protein kinase B) signaling axis are resistant to LeTx- or ERK inhibition-induced cell cycle arrest through protecting degradation and/or inducing expression of cyclin D1 [15,18,19]. However, the mechanism by which LeTx activates the adaptive PI3K-AKT signaling axis is yet to be elucidated.

In certain murine macrophages, LeTx rapidly induces pyroptosis, which is a programmed necrotic cell death mediated by the NACHT-leucine-rich repeat and pyrin domain-containing protein 1b and inflammasome [20]. These macrophages also undergo a similar adaptive response as in human monocytes and become resistant to LeTx-induced pyroptosis [5,21,22]. Our previous studies elucidated the adaptive response as an epigenetic phenomenon that silences mitochondrial cell death genes through the histone deacetylase (HDAC) 8 [23,24]. This study examined the role and mechanism of HDAC8 in adaptive responses of the human monocytic THP-1 cells to LeTx-induced cell cycle arrest. We found that HDAC8 played a pivotal role in activating the PI3K-AKT signaling axis in LeTx-exposed cells through inducing histone H3 lysine 27 tri-methylation (H3K27me3) and subsequent inhibition of PTEN expression.

## 2. Results

### 2.1. HDAC8 Decreases Susceptibility to LeTx-Induced Cytotoxicity

**Figure 1:**
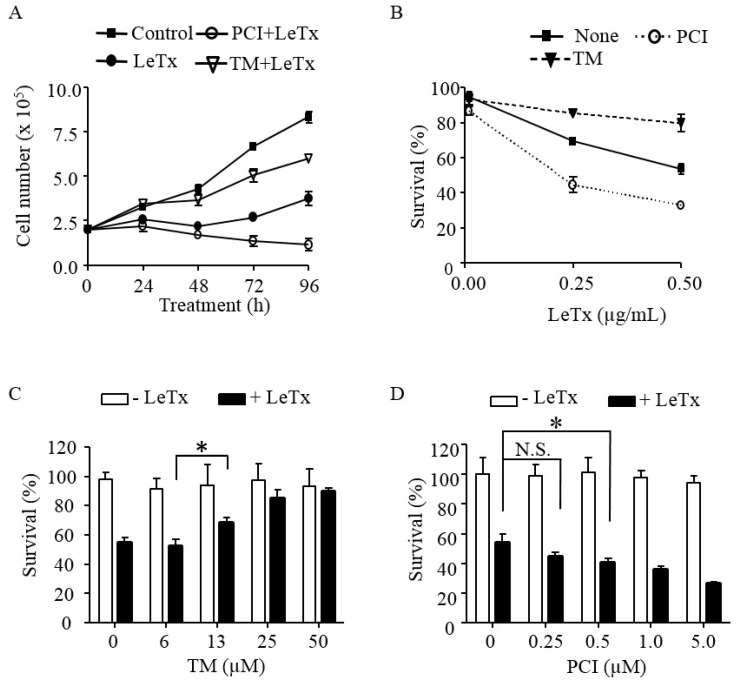
HDAC8 regulates susceptibility to LeTx cytotoxicity in human monocytic THP-1 cells. (**A**) THP-1 cells were treated with LeTx (500 ng/mL LF and 500 ng/mL PA) for 3 h and further cultured in fresh media in the presence or absence of TM-2-51 (TM; 25 µM) or PCI-34051 (PCI; 5 µM). Trypan blue staining negative live cells were counted using a hemocytometer at the time indicated. (**B**) THP-1 cells were treated with different doses of LeTx for 3 h and cultured in fresh media with or without TM (25 µM) and PCI (5 µM) for 48 h. Cell viability was measured using MTT assay. (**A**,**B**) Results are expressed as the mean ± SD (*n* = 3). (**C,D**) Similarly, cells were treated with LeTx in the presence or absence of various doses of: TM for 48 h (**C**); and PCI for 72 h (**D**). Cell viability was measured by MTT assay. Data are expressed as means ± SD (*n* = 3; N.S., not significant; *, *P* < 0.05, Student’s *t* test).

To examine the role of HDAC8 in cytotoxic effects of LeTx, THP-1 cells were treated with LeTx in the presence or absence of the HDAC8-specific activator TM-2-51 (TM) [25] or inhibitor PCI-34051 (PCI) [26], and total live cell numbers were counted each day for four days (Figure 1A). Cell numbers of LeTx-treated cells remained constant for 2–3 days and then started increasing on Day 3 (Figure 1A). Numbers of cells treated with both LeTx and PCI were steadily decreased throughout the experimental period (four days). However, cells treated with both LeTx and TM were continuously increased, albeit with a slower rate than that of non-treated cells. The cytotoxic and cytoprotective effects of PCI and TM, respectively, were similarly observed in two different doses of LeTx (Figure 1B). Significant cytoprotective effects of TM were detected, starting at 13 µM and maximum at 25 µM (Figure 1C). In contrast, PCI gradually increased cytotoxicity of LeTx, becoming statistically significant at 0.5 µM and maximally at 5 µM (Figure 1D).

### 2.2. HDAC8 Prevents Cell Cycle Arrest Induced by LeTx

**Figure 2:**
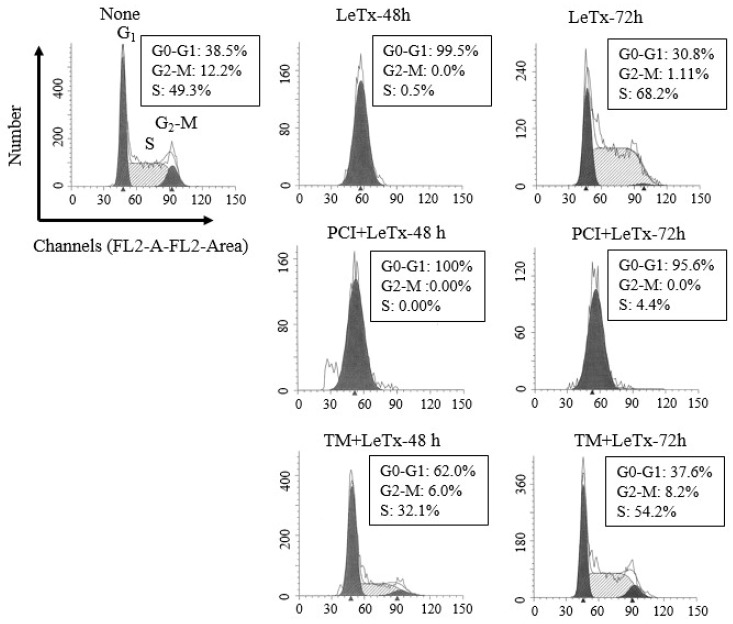
HDAC8 activity regulates susceptibility to LeTx-induced cell cycle arrest. THP-1 cells were treated with LeTx as described in the legend to Figure 1A. Cells were then harvested at 48 h or 72 h after LeTx treatments and fixed with 70% ethanol. Cell cycle phase was measured by the FACS Calibur flow cytometry/CellQuest program using propidium iodide DNA staining, followed by data analysis using ModFit software. Data shown are representative results of two independent experiments.

LeTx causes a transient cell cycle arrest in THP-1 cells [15]. Similarly, we detected a complete cell cycle arrest at G_o_-G_1_ stage 48 h post LeTx treatment, which was then spontaneously recovered 72 h post LeTx treatment (Figure 2; top lane). However, when cells were treated together with PCI, they failed to recover from the arrest (middle lane). Cell cycle progression of cells treated together with TM was slightly altered in 48 h, but became almost normal in 72 h of LeTx treatments (lower lane). These results indicate that HDAC8 protects cells from LeTx-induced cell cycle arrest.

### 2.3. HDAC8 Activity is Required for Activating AKT in LeTx-Treated Cells

**Figure 3:**
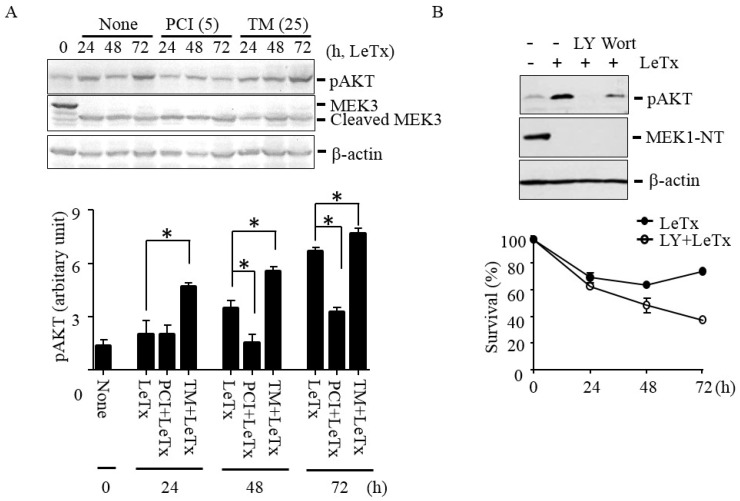
HDAC8 regulates AKT phosphorylation in THP-1 cells. (**A**) THP-1 cells were treated with LeTx as described in the legend to Figure 1A in the presence or absence of TM (25 µM) and PCI (5 µM). AKT phosphorylation at Ser-473 and MEK3 cleavage was analyzed using Western blots (upper panel). Western blotting against β-actin was used as the loading control. Results are representative blots from three independent experiments. Immunoreactivities against phospho-AKT (Ser-473) were analyzed using the NIH Image program (lower panel) and relative phosphor-AKT immunoreactivity was normalized to those of β-actin. Data are means and SD (*n* = 3; *, *P* < 0.05, Student’s *t* test). (**B**) Similarly, cells were treated with LeTx in the presence or absence of LY294002 (LY, 10 µM) or wortmannin (Wort, 1 µM) for 48–52 h. AKT phosphorylation and MEK1 N-terminal cleavage were measured by Western blotting (upper panel). Immunoblots shown are representative images of three independent experiments. For cytotoxicity assay, cells were similarly treated with LeTx in the presence or absence of LY (10 µM). Cytotoxicity was measured using MTT assay at the time points indicated (lower panel). Data are expressed as means and SD (*n* = 3).

Previously, we showed that activation of PI3K-AKT signaling axis prevents LeTx-induced cell cycle arrest [15]. To examine if HDAC8 is involved in the activation of AKT in LeTx-treated cells, we analyzed phosphorylation of AKT at the Serine 473 residue. AKT phosphorylation was increased in 24 h and peaked in 72 h after LeTx treatments (Figure 3A). In line with the previous results (Figure 1 and Figure 2), AKT phosphorylation was inhibited by PCI in 48 h post LeTx treatment; whereas, TM further enhanced AKT activation in 24 h after LeTx-treatment. The differences in AKT activation were not due to the levels of LeTx present in cells, since almost complete degradation of MEK3 was detected in all samples throughout the experimental time frame (Figure 3A, the 2nd lane). In addition, the AKT phosphorylation was inhibited by the PI3K inhibitors LY294002 (LY) and wortmannin (Wort; Figure 3B, upper panel), and LY further enhanced cytotoxic effects of LeTx (Figure 3B, lower panel). These results suggest that both HDAC8 and PI3K were involved in LeTx-induced AKT activation.

### 2.4. LeTx Suppresses PTEN Expression through HDAC8

**Figure 4:**
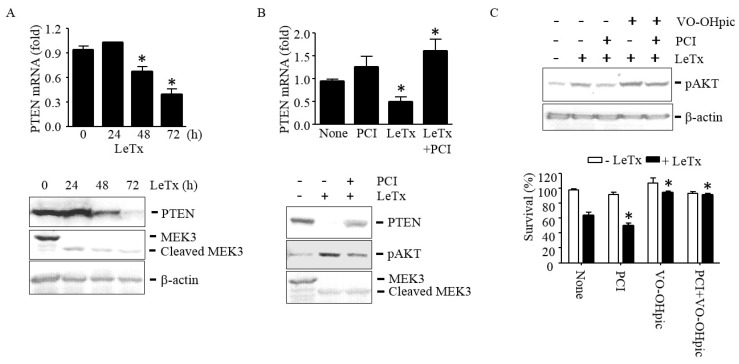
HDAC8 is required for suppression of PTEN expression in LeTx-exposed THP-1 cells. **(A–C**) Cells were treated with LeTx as described in the legend to Figure 1 in the presence or absence of PCI (5 µM) or VO-OHpic (10 µM) as indicated. (**A**) PTEN mRNA expression and protein levels were measured by qPCR (upper panel) and Western blotting (lower panel), respectively. (**B**) At 48 h after treatments, PTEN mRNA levels were measured by qPCR (upper panel), and PTEN protein and AKT phosphorylation levels were analyzed by Western blotting. (**C**) Similarly, AKT phosphorylation was measured by Western blotting (upper panel) and cell survival was measured by MTT assay (lower panel). Immunoblot images are representative of three independent experiments. Data shown are expressed as means ± SD (*n* = 3; *, *P* < 0.05, Student’s *t* test).

HDAC8 is an epigenetic modifier that regulates gene transcription. Among several signaling molecules known to regulate PI3K and AKT activation, expression levels of PTEN have shown to be inversely correlated with AKT activation in many cell types [27]. In THP-1 cells, LeTx suppressed PTEN expression in both mRNA and protein levels in 48 h after treatments (Figure 4A). PCI inhibited PTEN down-regulation and subsequently reduced AKT phosphorylation in LeTx-treated cells (Figure 4B). In line with these results, the PTEN-specific inhibitor VO-OHpic enhanced AKT phosphorylation (Figure 4C, upper panel) and cell proliferation (Figure 4C, lower panel) even in the presence of PCI in LeTx-treated cells. These results suggest that AKT activation in LeTx-treated cells was likely due to HDAC8-mediated PTEN down-regulation.

### 2.5. HDAC8 Induces Histone H3K27me3 by Silencing JMJD3

**Figure 5:**
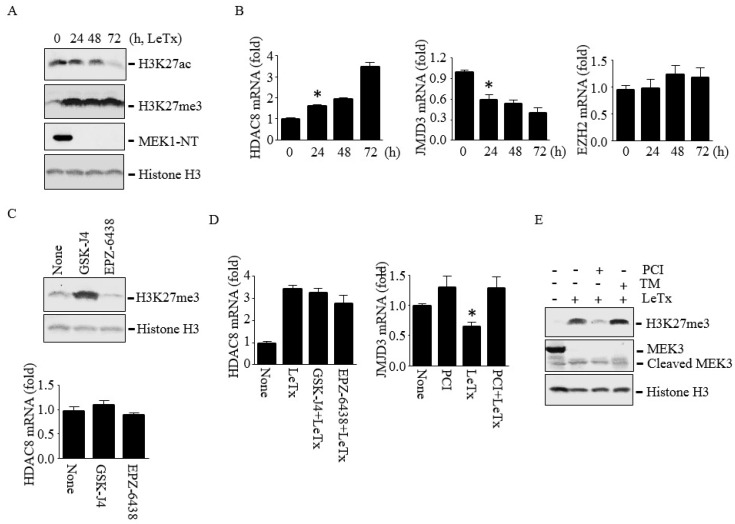
HDAC8 induces H3K27me3 through down-regulating JMJD3 in THP-1 cells. Cells were treated with LeTx as described in the legend to Figure 1 in the presence or absence of PCI (5 µM), GSK-J4 (1 µM) or EPZ-6438 (500 nM) for 24–72 h. (**A**) Levels of H3K27Ac and H3K27me3 were analyzed by Western blotting. Histone H3 immunoblot was used as the loading control. (**B**) HDAC8, JMJD3 and EZH2 mRNA levels were analyzed by qPCR. Results are expressed as means ± SD (*n* = 3). (**C**) Cells were treated with LeTx in the presence or absence of GSK-J4 or EPZ-6438 for 48 h. H3K27me3 (upper panel) and HDAC8 mRNA (lower panel) levels were analyzed by Western blotting and qPCR, respectively. (**D**) Similarly, cells were treated with LeTx, together with PCI or GSK-J4 or EPZ-6438 for 48h, and mRNA expression of HDAC8 (left panel) and JMJD3 (right panel) were measured by qPCR. Data are expressed as means ±SD (*n* = 3; *, *P* < 0.05, Student’s *t* test). (**E**) H3K27me3 levels in LeTx treated cell with or without PCI or TM were analyzed by Western blotting.

We previously showed that LeTx or HDAC8 overexpression leads to H3K27Ac deacetylation in murine macrophages [22,28]. Since the levels of H3K27Ac and H3K27me3 are inversely correlated, and an increase of H3K27me3 leads to down-regulation of PTEN expression [29,30,31,32], we examined both acetylation and methylation levels of H3K27. As expected, LeTx reduced H3K27Ac but induced H3K27me3 (Figure 5A). However, H3K27 methylation peaked in 24 h, which was faster than the rate of deacetylation that gradually occurred over 72 h of LeTx treatments. We then examined the expression levels of HDAC8, and the H3K27-specific methyltransferase enhancer of zeste 2 (EZH2)[33] and demethylase JMJD3 [34,35] in response to LeTx. LeTx had no effects on the expression levels of EZH2, but induced and inhibited, respectively, the expression of HDAC8 and JMJD3 (Figure 5B). To further examine if JMJD3 and EZH2 regulate HDAC8 expression or vice versa, THP-1 cells were treated with the JMJD3 inhibitor GSK-J4 and EZH2 inhibitor EPZ-6438. As expected, H3K27me3 levels were increased by GSK-J4 and decreased by EPZ-6438; however, these inhibitors had no effects on HDAC8 expression (Figure 5C). In addition, none of these inhibitors affected the expression levels of HDAC8 in LeTx-treated cells (Figure 5D, left panel). In contrast, PCI was able to prevent down-regulation of JMJD3 expression (Figure 5D, right panel) and subsequently decreased H3K27me3 levels (Figure 5E). These results suggest that HDAC8 regulates JMJD3 expression and H3K27me3 levels.

### 2.6. GSK-J4 Induces AKT Activation and Prevents Cell Cycle Arrest even in the Presence of PCI in LeTx-Treated Cells

**Figure 6:**
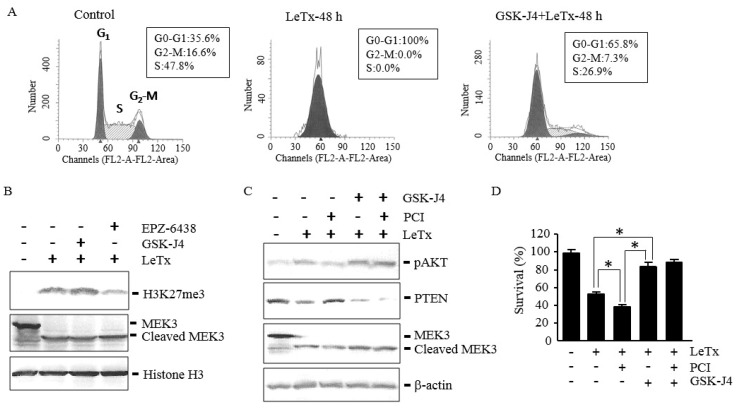
JMJD3 inhibition suppresses PTEN expression, enhances AKT phosphorylation and protects cell cycle arrest in LeTx-treated cells even in the absence of HDAC8 activity. THP-1 cells were treated with LeTx as described in the legend to Figure 1 in the presence or absence of PCI (5 µM), GSK-J4 (1 µM) or EPZ-6438 (500 nM) for 48 h. (**A**) Cell cycle analysis was performed as described in the legend to Figure 2. Results shown are representative data from three independent experiments. (**B,C**) H3K27me3, AKT phosphorylation at Ser-473 and PTEN protein levels were analyzed by Western blotting. Data shown are representative images of three independent experiments. (**D**) Cell viability was analyzed using MTT assay. Results are expressed as the mean ± SD (*n* = 3; *, *P* < 0.01, Student`s *t* test).

Since down-regulation of JMJD3 was a key step for increasing H3K27me3 levels in LeTx-treated cells, we examined if direct inhibition of JMJD3 influenced cell cycle arrest, PTEN expression and AKT activation in LeTx-treated cells. As expected, the JMJD3 inhibitor GSK-J4 attenuated cell cycle arrest in LeTx-treated cells (Figure 6A). GSK-J4 enhanced H3K27me3 (Figure 6B) and even outcompeted the PCI effects, resulting in decrease of PTEN expression and increase of AKT phosphorylation (Figure 6C). Importantly, GSK-J4 protected cell cycle arrest induced by LeTx even in the presence of PCI (Figure 6D).

## 3. Discussion

Here, we demonstrated that HDAC8 played a key role in determining susceptibility to cell cycle arrest induced by LeTx. TM, which increases HDAC8 activity up to 12-fold at 10 µM concentration [25], significantly prevented cell cycle arrest induced by LeTx at 13 µM and maximally at 25 µM concentrations. In contrast, PCI further enhanced the duration and extent of cell cycle arrest elicited by LeTx in a dose-dependent manner. In line with these observations, AKT phosphorylation in LeTx-exposed cells was further enhanced by TM, but was inhibited by PCI. Phosphorylation of AKT at Ser-473 is essential for its full activation and stabilization of active conformation [36,37], and mediated by the mammalian target of rapamycin complex 2 and other PI3K-independent kinases [38,39,40]. However, LeTx likely induced AKT phosphorylation through a PI3K-dependent pathway, since the PI3K inhibitors LY and wortmannin inhibited AKT phosphorylation and cell proliferation.

PTEN is a tumor suppressor gene that counteracts PI3K activity through dephosphorylating PI(3,4,5)P_3_ to produce PI(4,5)P_2_ [41]. PTEN activity is regulated by multiple post-translational modifications such as phosphorylation, ubiquitylation, acetylation, SUMOylation, oxidation and protein–protein interactions, but transcriptional regulation of PTEN plays a key role in determining PI3K-AKT dependent cell proliferation, survival and metabolic phenotypes [27,42,43,44]. Various cellular stresses and growth factors also regulate expression of PTEN [45,46,47,48]. Activation of MAPKs is involved in the regulation of PTEN expression; however, its role in PTEN expression varies depending on cell types [48,49,50]. We detected PTEN mRNA and protein expression levels were down-regulated by LeTx (Figure 4A) in a PCI-sensitive manner (Figure 4B). Consistently, the PTEN-specific inhibitor VO-OHpic [51] further enhanced AKT phosphorylation and cell survival in LeTx-treated cells. Collectively, these results suggest that LeTx induces AKT activation at least in part through HDAC8-dependent down-regulation of PTEN expression.

HDAC8 has shown to be involved in regulating cell cycle progression of normal and tumor cells [52]. Overexpression of HDAC8 has been associated with cell proliferation of multiple cancer cells [53]; whereas, defective mutations of HDAC8 are linked to Cornelia de Lange syndrome, which is a rare genetic disease manifesting congenital malformations of multiple organs due to defects in cohesin [52]. Cohesin is a multi-protein complex which forms a clutch to hold the sister chromatids together during S phase [54]. HDAC8 deacetylates the cohesin subunit Structural Maintenance of Chromosomes 3 (SMC3) at lysines 105 and 106, resulting in segregation of cohesion from the sister chromatids and replenishes SMC3 for another cell cycle. Inhibition of HDAC8 leads to accumulation of acetylated SMC3, and consequently cell cycle arrest [55]. For example, in breast tumor MCF7 cells and neuronal fibroblasts, inhibition or defects in HDAC8 prolongs G_1_ phase and delays entering to S phase [56,57]. Similarly, PCI further enhanced cell cycle arrest at G_0/1_ phase in LeTx-treated cells, which could be an additional cell cycle arrest mechanism induced by PCI. However, PCI alone had no effects on cell cycle progress within our experimental time frame and TM was able to prevent cell cycle arrest in LeTx-treated cells, suggesting that HDAC8 regulates cell proliferation through regulating PI3K-AKT signaling axis induced by LeTx, independent of SMC3 acetylation. However, further studies are required to rule out the involvement of SMC3 in HDAC8-mediated cell cycle regulation in LeTx-treated cells.

Previously, several studies have shown that the Polycomb Repressive Complex 2 (PRC2), which writes and reads H3K27me3, suppresses gene expression including PTEN in various cell types [32,58,59,60,61,62,63]. It is also known that H3K27 acetylation is mutually exclusive to methylation of the residue and H3K27 deacetylation is a prerequisite chromatin context for PRC2 to be active on histones [29,30,31]. In murine macrophages, we showed that LeTx suppresses gene transcription in part through up-regulating HDAC8 that leads to H3K27 deacetylation [23,28]. As expected, LeTx also induced HDAC8 mRNA expression, decreased H3K27Ac levels and increased H3K27me3 levels over 3 days in THP-1 cells. Apparently, increase of H3K27me3 levels was at least in part due to down-regulation of the H3K27 demethylase JMJD3. LeTx decreased JMJD3 expression but not EZH2 which is a subunit of PRC2 with H3K27 methyltransferase activity. PCI was able to prevent down-regulation of JMJD3 and prevent H3K27me3; whereas, the JMJD3- and EZH2-specific inhibitors GSK-J4 and EPZ-6438 had no effects on HDAC8 expression in LeTx-treated cells. However, we could not detect a direct inverse-correlation in the levels of H3K27Ac and H3K27me3. H3K27Ac levels were gradually down-regulated over 3 days, but H3K27me3 levels were rapidly and maximally increased in 24 h after LeTx treatments. At this moment, we could not rule out the involvement of other HDAC8-dependent signaling events in rapidly inducing H3K27me3. However, GSK-J4 suppressed PTEN expression, activated AKT phosphorylation and prevented cell cycle arrest even in the presence of PCI in LeTx-treated cells, supporting a crucial role of JMJD3 in inducing H3K27me3 and PTEN suppression. HDAC8 has also been shown to regulate transcription through targeting non-histone proteins, such as adenoviral E1A-12 protein [64], the inversion-16 fusion gene products in acute myeloid leukemia cells [65], p53 [66], protein phosphatase (PP) 1 [67], heat shock proteins [68], α-actin [69], human ever-shorter telomeres 1B [70], estrogen-related receptor-α [71] and possibly multiple other proteins [72,73]. Of interest, HDAC8 deacetylates and activates p53 [66]. Since p53 binds to the PTEN promoter and enhances PTEN gene transcription [74], it is possible that HDAC8 induces PTEN expression by activating p53. To date, it is unknown whether LeTx induces cell cycle arrest and/or cell death through activating p53. Therefore, the involvement of p53 in preventing LeTx-induced cell cycle arrest by HDAC8 warrants further studies.

**Figure 7:**
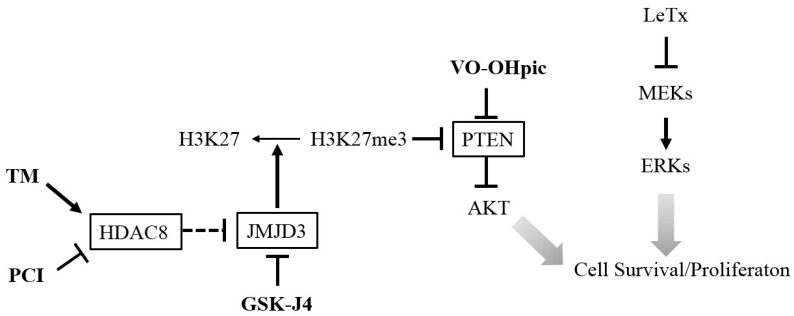
A diagram illustrating the targets, and proposed effects on AKT activation and cell survival/proliferation of the inhibitors and activator. The HDAC8 activator TM and JMJD3 inhibitor GSK-J4 lead to an increase of H3K27me3 that suppress PTEN expression and enhances AKT activation. Direct inhibition of PTEN by VO-OHpic also induces AKT activation. In contrast, inhibition of HDAC8 by PCI leads to expression of PTEN and inhibits AKT activation. Activation of AKT promotes cell survival/proliferation in LeTx-exposed monocytes/macrophages. Arrows indicate a positive effect or activation and blocked arrows indicate a negative effect or inhibition.

Overall, this study showed that the HDAC8-JMJD3-H3K27me3-PTEN-AKT signaling axis played a key role in determining susceptibility to cell cycle arrest in LeTx-intoxicated cells (Figure 7). Activation of the signaling axis by either the HDAC8 activator TM, JMJD3 inhibitor GSK-J4 or PTEN inhibitor VO-OHpic induced AKT activation and prevented cell cycle arrest. In contrast, the HDAC8 inhibitor PCI suppressed AKT activation and potentiated LeTx-induced cytotoxicity. Macrophages play a complex role in the pathogenesis of anthrax, involved in both systemic dissemination of and innate immunity to *B. anthracis* [75,76]. LeTx is released by germinating spores within macrophage phagosomes at an early stage of infection [77] and systemic vegetative bacilli at a later stage, facilitating bacterial dissemination [75,78] and immune paralysis, respectively [75,76]. Particularly, generation and survival of macrophages were suggested to be important for preventing anthrax at a later stage [4,79]. Therefore, we speculate that therapeutic approaches either inhibiting HDAC8 before systemic dissemination or activating HDAC8 and inhibiting JMJD3 at a later stage could be novel strategies for inhibiting dissemination or maintaining macrophage populations during *B. anthracis* infection. Interestingly, modified anthrax lethal toxins, which cleave and inactivate MEKs selectively in tumors, is an emerging anti-tumor biomolecule with promising results [80]. However, using LeTx is expected to be limited in its efficacy due to adaptive responses often observed in various tumors. Therefore, targeting HDAC8 could also be a novel strategy for developing combinatory anti-tumor therapies.

## 4. Materials and Methods

### 4.1. Reagents

PA and LF were purchased from the List Biological Laboratories (Campbell, CA, USA). Chemical inhibitors used in this study are the following: PCI-34051(Cayman chemical, Ann Arbor, MI, USA), TM-2-51(1-Benzoyl-3-phenyl-2-thiourea, Sigma-Aldrich, St.Louis, MO, USA), JMJD3 inhibitor GSK-J4 (Sigma-Aldrich), EZH2 inhibitor EPZ-6438 (MedChemexpress CO., Ltd; Princeton, NJ, USA, through CEDARLANE), PI3K inhibitors LY294002 (ApexBio Technology, Houston, TX, USA) and wortmannin (Calbiochem, La Jolla, CA, USA), PTEN inhibitor VO-OHpic trihydrate (BioVision, Milpitas, CA, USA). Propidium iodide and RNase A were obtained from Calbiochem and Sigma-Aldrich.

Antibodies raised against the NH_2_-terminus of MEK1 (MEK1-NT) and MEK3 were obtained from Stressgen Bioreagents (Ann Arbor, MI, USA) and Santa Cruz Biotechnology (Dallas, TX, USA), respectively. The phospho-AKT (Ser-473) and PTEN antibodies were purchased from Cell Signaling and Cedarlane (Danvers, MA, USA and Burlington, NC, USA). Anti- H3K27Ac and anti-H3K27me3 antibodies were from Active Motif (Carlsbad, CA, USA); pan-histone H3 antibodies from Bio Vision; β-actin antibodies from Rockland Inc (Gilbertsville, PA, USA).

### 4.2. Cell Culture

The human monocytic cell line THP-1 cells were purchased from the American Type Culture Collection and cell cultures were maintained in complete RPMI 1640 medium containing 10% heated-inactivated fetal bovine serum (Sigma), 10 mM MEM non-essential amino acids solution, 100 U/mL penicillin G sodium, 100 μg/mL streptomycin sulfate and 1 mM sodium pyruvate as previously described [24].

### 4.3. Cell Viability Assay

Living cells in Figure 1A were counted using a hemocytometer after trypan blue staining. MTT (3-(4,5-dimethylthiazol-2-yl)-2,5-diphenyltetrazolium bromide) assay was also used for cell viability assay as described in previous studies [24]. Briefly, THP-1 cells were cultured in 96 well plates and treated with LeTx with or without subsequent treatments with chemical reagents for the time indicated. MTT was then added at a final concentration of 0.5 mg/mL, and incubated at 37 °C for an additional 2 h. Culture media was aspirated and 100 µL of dimethyl sulfoxide was added to dissolve crystals. Optical densities of each well were analyzed at OD_570 nm_ using a microplate reader (Synergy H4 Hybrid Reader; BioTek Instruments Inc., Winooski, VT, USA). The percentage of cell survival was estimated based on OD_570 nm_ of wells by comparing those from non-treated cells.

### 4.4. Western Blot

Preparation of total cell lysates and immunoblotting were performed as previously reported [23]. Briefly, cells were lysed in ice-cold lysis buffer containing 20 mM MOPS, 2 mM EGTA, 5 mM EDTA, 1 mM Na_3_VO_4_, 40 mM β-glycerophosphate, 30 mM sodium fluoride, 20 mM sodium pyrophosphate, 0.1% SDS, 1% Triton X-100, pH 7.2, and a protease inhibitor cocktail (Roche, Werk Penzberg, Germany). Cell lysates were collected after centrifugation at 12,500 rpm for 15 minutes at 4 °C. Proteins were then separated by SDS-polyacrylamide gels and transferred onto nitrocellulose membranes (Bio-Rad). The membranes were blocked with 5% (*w*/*v*) skim milk for 1 h and incubated overnight at room temperature with primary antibodies. After washing three times with 1 × TTBS (20 mM Tris, 150 mM NaCl, pH 7.5) containing 0.07% tween 20, the membranes were incubated with secondary antibodies for 1 hour at room temperature and films were developed using an enhanced chemiluminescence detection system (ECL; Thermo Scientific, Waltham, MA, USA).

### 4.5. Quantitative Real-Time PCR

mRNA expression was quantified by quantitative real-time PCR (qPCR) as previously described [23]. Briefly, total cellular RNAs were isolated using TRIzol (Ambion by Life Technologies, Carlsbad, CA, USA) according to the manufacturer's instructions and mRNAs were reverse transcribed using Moloney murine leukemia virus (M-MuLV) reverse transcriptase (New England Biotechnology, Ipswich, MA, USA) and oligo (dT) as a primer. Quantitative real-time PCR (qPCR) analyses were performed with a Rotor-Gene RG3000 quantitative multiplex PCR instrument (Montreal Biotech Inc, Dorval, QC, Canada) using Power UP^TM^ SYBRR Green Master Mix (Applied Biosystems life technologies, Foster City, CA, USA). The data were normalized by expression of the GAPDH housekeeping gene. Primers used for qPCR are listed in the following; for GAPDH, 5’-ACCCACTCCTCCACCTTTG-3’ (5’ primer) and 5’-CTCTTGTGCTCTTGCTGGG-3’ (3’ primer); for PTEN, 5’-ACTTGCAATCCTCAGTTTGTGG-3’ (5’ primer) and 5’-GAAGAATGTATTTACCCAAAAGTG-3’ (3’ primer); for HDAC8, 5’-ATTCTCTACGTGGATTTGGATC-3’ (5’ primer) and 5’-ATGCCATCCTGAATGGGCACA -3’ (3’ primer); for JMJD3, 5’-TCTGATGCTAAGCGGTGGAAG-3’ (5’ primer) and 5’-GCCAATGTTGATGTTGACGGAG-3’ (3’ primer); for EZH2, 5’-GTGCCATTGCTAGGTTAATTGG-3’ (5’ primer) and 5’-AGGGTTGATAGTTGTAAACATGG-3’ (3’ primer).

### 4.6. Cell Cycle Analysis

Analyses of DNA content were performed using propidium iodide (PI) and Cell Quest software on a FACS calibur flow cytometer (Becton Dickinson Biosciences, San Jose, CA, USA) as previously reported [15]. Briefly, 1.0 × 10^6^ cells were harvested and fixed by drop-wise an addition of ice-cold 70% ethanol after washing three times with 1 × PBS containing 0.1% glucose, and cells were then stored at 4 °C until processing PI staining. Subsequently, cells were pelleted by centrifugation and re-suspended in PI staining solution (0.1% glucose in 1 × PBS) containing 50 µg of PI/mL and 100 unit of RNase A/mL. After two hours of incubation at room temperature, the cell was loaded onto a FACS calibur flow cytometer. Data were acquired and analyzed using Cell Quest and ModFit LT 3.0 software (Becton Dickinson, San Jose, CA, USA).

### 4.7. Statistical Analysis

Data were analyzed using GraphPad Prism 4.0 (GraphPad Software Inc., San Diego, CA, USA). The results are presented as the means ± SD of three independent repeats. Statistical significance was defined as *p* < 0.05 (*). Otherwise, it was mentioned in the figure legends.

## Abbreviations

ERK	extracellular signal-regulated kinase
EZH2	enhancer of zeste 2
HDAC	histone deacetylase
H3K27Ac	histone H3 lysine 27 acetylation
H3K27me3	histone H3 lysine 27 tri-methylation
JMJD3	Jumonji Domain Containing 3
LeTx	anthrax lethal toxin
LF	lethal factor
LY	LY942006
MAPK	mitogen-activated protein kinase
MEK	MAPK kinase
MTT	3-(4,5-dimethylthiazol-2-yl)-2,5-diphenyltetrazolium bromide
PA	protective antigen
PI3K	phosphoinositide 3-kinase
SMC3	structural maintenance of chromosomes 3
PCI	HDAC8 inhibitor PCI34051
PTEN	phosphatase and tensin homolog 1
TM	HDAC8 activator TM-2-51
